# Electroencephalography-based Alzheimer's disease detection using multi-scale information retention transform: a subject-level comparison of machine learning classifiers

**DOI:** 10.3389/fneur.2026.1869211

**Published:** 2026-09-30

**Authors:** Peng Zhang, Wei Tong, Lijun Jiang, Jing Kan, Wenjie Zeng, Bicheng Wu, Haisheng Zhang

**Affiliations:** 1 Affiliated Xiaoshan Hospital, Hangzhou Normal University, Hangzhou, Zhejiang, China; 2 Laboratory of Intelligent Brain Neuroimaging, Advanced Institute of Information Technology (AIIT), Hangzhou, Zhejiang, China; 3 School of Statistics and Data Science, Zhejiang Gongshang University, Hangzhou, Zhejiang, China; 4 Department of Clinical Psychology, Affiliated Hangzhou First People's Hospital, Westlake University School of Medicine, Hangzhou, Zhejiang, China

**Keywords:** Alzheimer's disease, electroencephalography, entropy, feature fusion, machine learning

## Abstract

**Introduction:**

This exploratory study evaluates Multi-Scale Information Retention Transform (MSIRT), a feature-fusion framework for electroencephalography (EEG)-based Alzheimer's disease (AD) classification.

**Methods:**

Ratios between differential entropies were removed, and all EEG amplitudes were converted to volts before feature extraction because the transform is not invariant to arbitrary physical units. Revised MSIRT combines fuzzy entropy (FE), permutation entropy (PE), and differential entropy (DE) using training-fold normalization and cross-feature products. The framework was evaluated on OpenNeuro ds004504 (29 healthy controls and 36 participants with AD) using participant-level cross-validation.

**Results:**

With Random Forest (RF) held fixed, direct concatenation achieved 84.62% accuracy and an area under the receiver operating characteristic curve (AUC) of 0.8956, whereas revised MSIRT achieved 86.15% accuracy and an AUC of 0.9004. An interaction-only control achieved 80.00% accuracy and an AUC of 0.8908. Because the no-product transform is feature-wise strictly monotonic, it was treated as an RF invariance control rather than a source of new predictive information. The paired AUC difference between revised MSIRT and concatenation was 0.0048 (bootstrap 95% confidence interval, −0.0067 to 0.0192; paired permutation *p* = 0.3780). In 20 sex-balanced resamples, revised MSIRT had a mean AUC of 0.8846.

**Discussion:**

These results do not establish statistical superiority or clinical utility.

## 1 Introduction

Alzheimer's disease (AD) is a common progressive neurodegenerative disorder and an increasing public-health burden in aging populations (1). Clinical manifestations and rates of progression vary between individuals, which complicates assessment across the disease course (1). These challenges motivate research into objective and repeatable measurements that may complement established clinical assessments.

Clinical evaluation combines history, cognitive assessment, and neurological examination, with imaging or biofluid biomarkers used when appropriate (2). Each modality provides different information and has distinct constraints on cost, accessibility, or invasiveness (3, 4). Electroencephalography (EEG) is therefore being investigated as a non-invasive research modality that directly measures time-varying electrophysiological activity. It is not proposed here as a replacement for established diagnostic evaluation. Figure 1 compares established clinical methods with the proposed EEG-based research approach.

**Figure 1:**
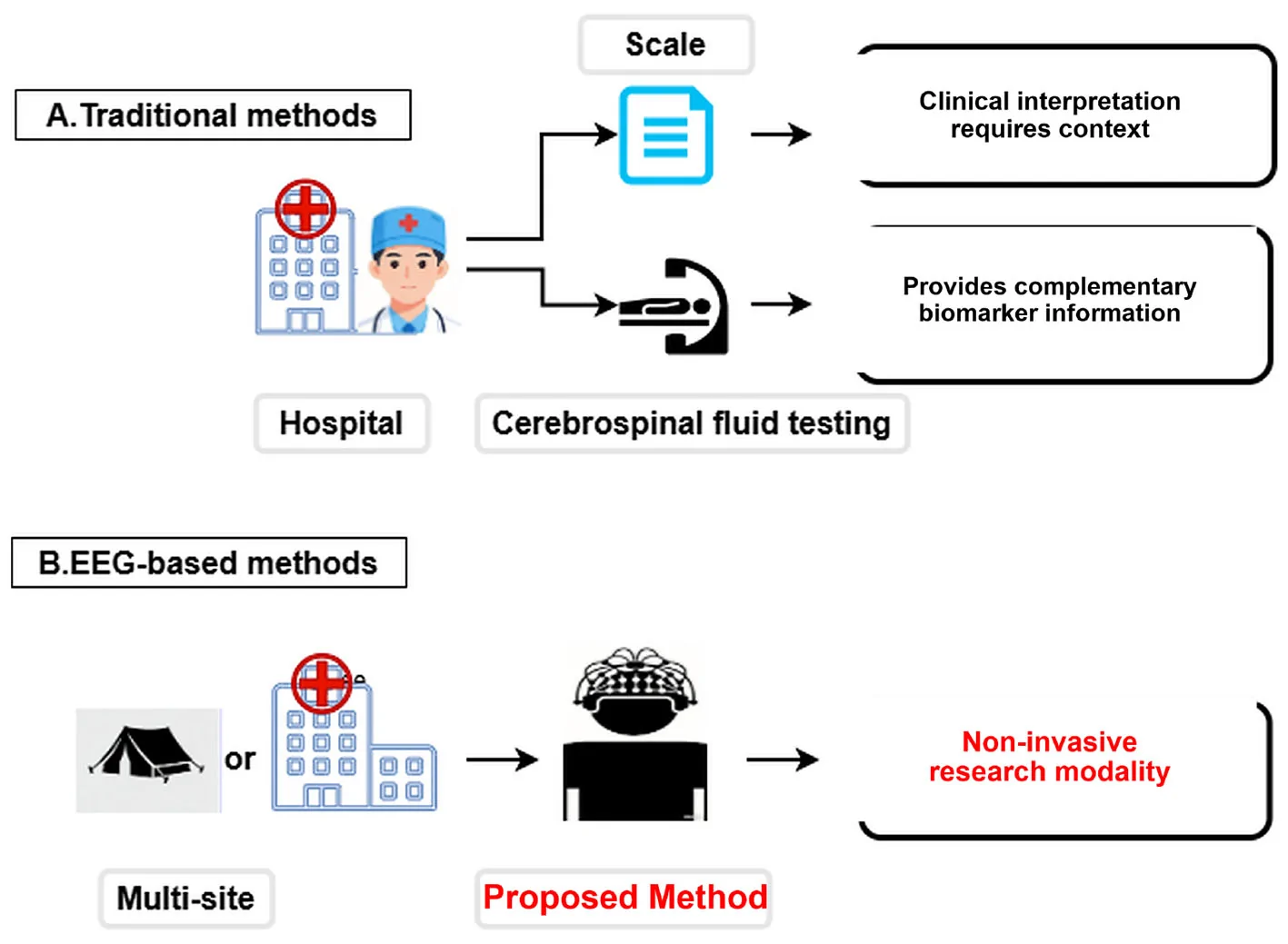
Comparison of established clinical methods and the proposed EEG-based research approach. The EEG workflow is evaluated as a non-invasive research modality rather than an established clinical replacement.

EEG non-invasively records brain electrophysiological activity with high temporal resolution (5). AD studies have reported changes in spectral composition, synchrony, and signal complexity, although findings vary with cohort and methodology (5, 6). Resting-state EEG has consequently been investigated in statistical and machine-learning studies of AD (7). These observations motivate EEG classification research but do not by themselves establish a clinical screening application.

Non-linear analysis provides quantitative descriptions of EEG temporal complexity and irregularity. Original AD studies reported group differences using single-scale and multiscale entropy analysis of resting EEG (8, 9). Later work has examined fuzzy entropy (FE) and permutation entropy (PE) variants, while emphasizing sensitivity to parameters, amplitude scaling, pre-processing, and recording conditions (10–13).

FE quantifies time-series complexity using a fuzzy membership function (14). AD EEG studies have applied FE and multiscale FE to assess group differences in signal complexity (10, 12). These studies also show that FE estimates depend on parameters and amplitude scaling.

PE quantifies the distribution of local ordinal patterns (15). PE-based analysis has identified reduced EEG complexity in AD and mild cognitive impairment at the group level (11).

Differential entropy (DE) extends Shannon entropy to continuous random variables. Under the Gaussian approximation used here, band-specific DE is a logarithmic function of the variance of the band-limited signal (16). This citation supports the feature definition rather than an AD-specific clinical claim.

Resting-state EEG classification studies have evaluated multiple entropy descriptors, but reported performance depends strongly on the cohort and validation design (7). The present study evaluates whether a transformation and explicit interaction terms alter performance relative to direct concatenation. Multi-Scale Information Retention Transform (MSIRT) integrates FE, PE, and DE from multichannel EEG signals through scale transformation and cross-feature terms. Five fixed classifiers are evaluated under the same participant-level protocol, and the fusion components are assessed separately with Random Forest (RF).

## 2 Materials and methods

### 2.1 Materials

This study used the publicly available OpenNeuro ds004504 dataset (17), which contains scalp EEG recordings from participants with AD, frontotemporal dementia, and healthy controls (HC). We selected the AD and HC groups, comprising 65 participants (29 HC and 36 AD). The HC group included 18 males and 11 females, with an age range of 57–78 years and a mean age of 67.9 ± 5.4 years. The AD group included 12 males and 24 females, with an age range of 49–79 years and a mean age of 66.4 ± 7.9 years. According to the original dataset descriptor, the recruiting neurologists established the clinical diagnoses using DSM-III-R, DSM-IV, or ICD-10 criteria, with AD additionally assessed according to the NINCDS–ADRDA guidelines (17). Cognitive status was evaluated using the Mini-Mental State Examination (MMSE). We did not independently adjudicate or modify the diagnoses supplied with the dataset. The source report provides the medication and exclusion criteria (17). No significant mean age difference was detected (*p* = 0.38), but this result does not exclude residual age-related confounding. The sex distribution differed between groups (two-sided Fisher's exact *p* = 0.0262), and this imbalance was examined in a pre-specified sensitivity analysis. EEG was recorded during eyes-closed rest from 19 scalp channels at 500 Hz. The participant characteristics are summarized in Table 1.

**Table 1:** Participant characteristics.

Subjects	Male	Female	Age (mean ±SD)
HC (*n* = 29)	18	11	67.9 ± 5.4
AD (*n* = 36)	12	24	66.4 ± 7.9

SD denotes standard deviation.

HC denotes healthy controls; AD denotes Alzheimer's disease. Diagnoses were supplied by the source dataset; AD was assessed according to DSM/ICD criteria and NINCDS–ADRDA guidelines. The groups differed in sex distribution (two-sided Fisher's exact *p* = 0.0262), whereas no significant mean age difference was detected (*p* = 0.38).

Figure 2 shows illustrative 500-Hz EEG segments from one HC and one AD participant. These examples are provided only to show the signal format and were not used as evidence of a group-level waveform difference.

**Figure 2:**
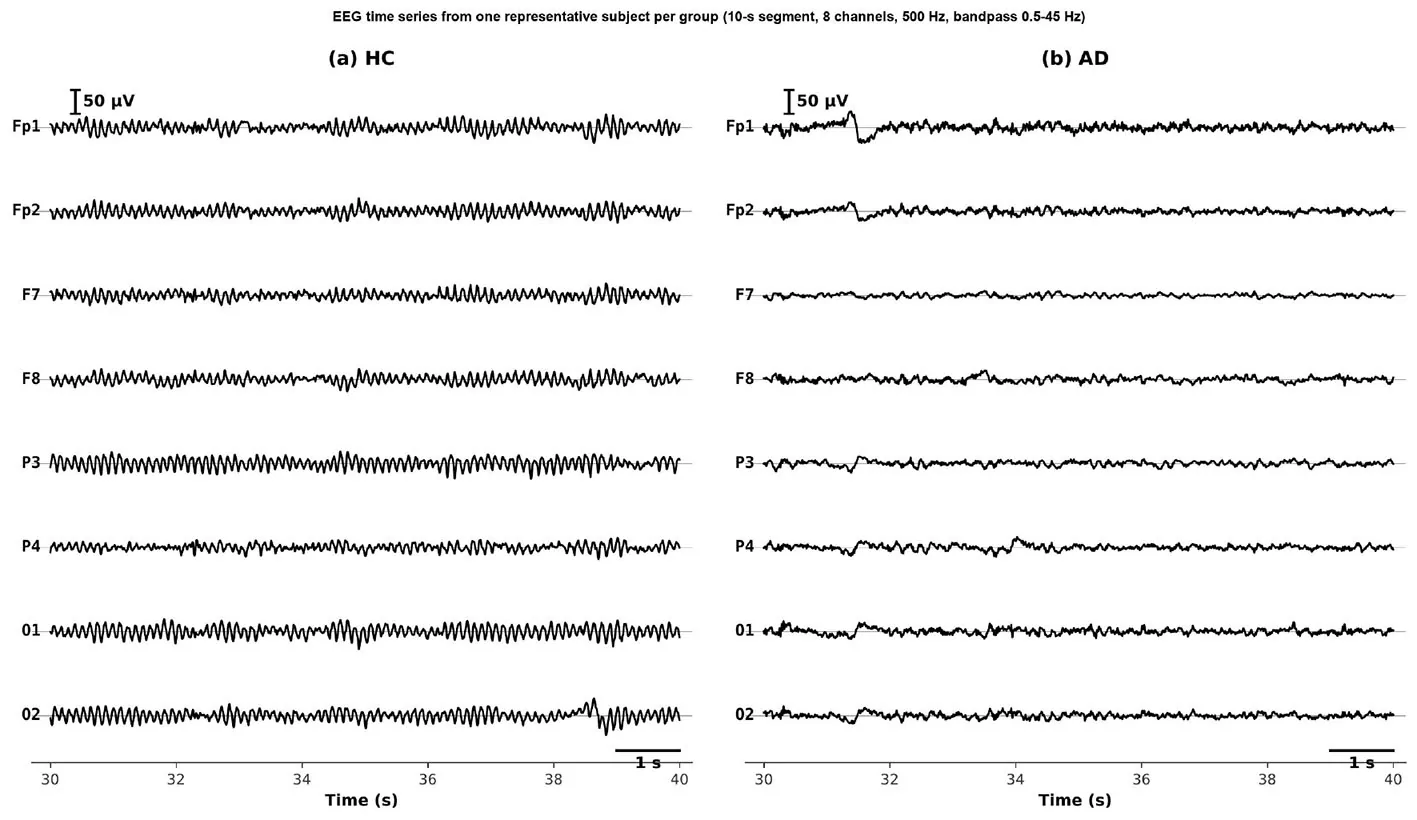
Illustrative 10-s, 500-Hz EEG segments from one participant in each group: **(a)** HC and **(b)** AD.

### 2.2 Methods

The analysis comprised four stages: loading the dataset-provided pre-processed derivatives, selecting channels and applying residual quality checks, extracting entropy features, and evaluating participant-level classification. Figure 3 summarizes this workflow. All study-specific operations are described below.

**Figure 3:**
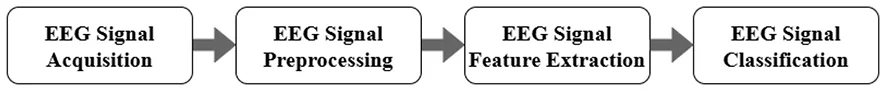
The pipeline of EEG-based AD detection model.

#### 2.2.1 Data acquisition and pre-processing

The EEG data were obtained from OpenNeuro ds004504, version 1.0.0 (OpenNeuro, Stanford Center for Reproducible Neuroscience, Stanford, California, United States) (dataset digital object identifier: 10.18112/openneuro.ds004504.v1.0.0) (17), accessed in July 2026. We analyzed the dataset-provided pre-processed EEGLAB derivative files and did not reprocess the raw Brain Imaging Data Structure (BIDS) recordings. The local analysis files followed dataset/{AD,HC}/sub-XXX_task-eyesclosed_eeg.set. According to the dataset documentation, pre-processing comprised a 0.5–45 Hz Butterworth band-pass filter, A1–A2 rereferencing, Artifact Subspace Reconstruction (ASR), RunICA, and ICLabel-guided removal of eye and jaw components. The supplied derivatives contained 19 channels sampled at 500 Hz.

The study-specific pipeline selected Fp1, Fp2, F7, F8, P3, P4, O1, and O2 directly from the supplied derivatives. Values stored in microvolts were converted to volts before quality control and feature extraction. This conversion defines the required physical unit for the implementation and was applied identically to every participant. No additional rereferencing, filtering, notch filtering, resampling, bad-channel interpolation, Artifact Subspace Reconstruction, or independent-component analysis was performed. In particular, no average reference was calculated either before or after selecting the eight channels.

The data were then divided into non-overlapping 2-s epochs. Residual quality control excluded epochs containing nonfinite values, an absolute amplitude of at least 500μV, or any selected channel with an epoch standard deviation below 0.5μV. Sustained high-noise epochs were also excluded when any channel's root-mean-square amplitude exceeded the recording-specific median by eight robust standard deviations. The robust standard deviation was defined as 1.4826 times the median absolute deviation across epochs. These rules screened residual extreme, flat, and persistently noisy segments after the dataset-provided artifact-component removal. They did not constitute new ocular or muscle-component correction. No additional bad-channel interpolation or independent-component analysis was performed, and no participant was removed.

For the primary analysis, 50 valid epochs were selected deterministically for each participant. The indices were generated as numpy.linspace(0, n_valid-1, 50, dtype=int), producing temporal spacing from the first to last valid epoch without a random seed. Every classifier and ablation used the same indices. Sampling sensitivity was assessed using 20 random 50-epoch selections with seeds 42–61 and a separate analysis using all quality-controlled epochs. The participant splits, model settings, and subject-level aggregation were held fixed across these comparisons.

For each epoch, three types of nonlinear entropy features were computed:

- Fuzzy Entropy (FE) with embedding dimension *m* = 2, similarity tolerance *r* = 0.2 (scaled by signal standard deviation), and fuzzy exponent *n* = 2.
- Permutation Entropy (PE) with embedding dimension *m* = 3 and time delay τ = 1. PE was normalized to the range [0, 1] by dividing by log(*m*!).
- Differential Entropy (DE) computed in five frequency bands: delta (0.5–4 Hz), theta (4–8 Hz), alpha (8–13 Hz), beta (13–30 Hz), and gamma (30–45 Hz). For each 2-s epoch and channel, power spectral density was estimated at the native 500-Hz sampling rate using Welch's method with a 256-sample segment, 128-sample overlap, and density scaling. Band power was obtained by trapezoidal integration of the spectral density over the corresponding inclusive frequency interval and divided by the band width to give the band-specific mean-power estimate $\hat{\sigma_{b}^{2}}$. Under the Gaussian approximation, $\text{D}{\text{E}}_{b}=0.5\log\left(2\pi e\max\left(\hat{\overset{2}{\underset{b}{\sigma }}},10^{-12}\right)\right)$, using the natural logarithm.

#### 2.2.2 Theoretical initials

FE, PE, and DE provide distinct mathematical descriptions of signal complexity, ordinal structure, and continuous-valued variability. The definitions used in this study are provided in Equations 1–3.

• The FE for the signal ***X***^*N*^ = {*x*(1), *x*(2), …, *x*(*N*)} can be calculated as follows:

$$\begin{array}{l} FE\left(m,r,n\right)=-\ln\text{[}\frac{\frac{1}{N-m+1}\sum_{i=1}^{N-m+1}B_{i}^{m+1}\left(n,r\right)}{\frac{1}{N-m}\sum_{i=1}^{N-m}B_{i}^{m}\left(n,r\right)}\text{]} \end{array}$$ (1)

where, *m* represents the length of the comparison vector, *r* represents the measure of similarity. *n* is the fuzzy exponent, which adjusts the decay rate of the fuzzy membership function to balance the noise immunity and sensitivity. And the $B_{i}^{m}\left(n,r\right)$represents the average similarity of the *m*-dimensional vector *X*^*m*^(*i*) reconstructed from the original sequence.

• The PE can be calculated as follows:

$$\begin{array}{l} PE\left(m,\tau \right)=-\overset{K}{\underset{j=1}{\sum }}P_{j}\ln P_{j} \end{array}$$ (2)

where, *m* is embedding dimension, characterizing the local dynamic dimension of the signal. τ represents the time delay, *K* is the total number of permutation patterns of *m*-dimensional embedded vectors, *P*_*j*_ Probability of the *i*-th permutation pattern.

• DE is an extension of Shannon entropy on continuous random variables and is mainly used to describe the amount of information of continuous random variables. Considering the signal as a continuous random variable, its DE can be calculated as follows:

$$\begin{array}{l} DE=-\overset{b}{\underset{a}{\int }}p\left(x\right)\log\left(p\left(x\right)\right)\text{dx} \end{array}$$ (3)

Here, *p*(*x*) represents the probability density function of the continuous variable, and [*a, b*] represents the value range of the variable.

#### 2.2.3 The proposed MSIRT algorithm

MSIRT addresses scale differences between FE, PE, and DE by combining logarithmic compression with robust normalization. It then adds explicit cross-feature products before classification. The following equations define the revised implementation. For any single feature *x* in the fused features, the calculation is as follows:


$$\begin{array}{l} \begin{array}{c} f\left(x\right)=W\times g\left(x\right)+\left(1-W\right)\times h\left(x\right)+k \end{array} \end{array}$$ (4)

Among them, *W* represents the scale information weight, *g*(*x*) is the macroscopic scale transformation function, *h*(*x*) is the microscopic scale transformation function, and *k* is the bias term. In this study, the bias term (*k*) is 0.

The MSIRT transformation is applied per feature type (FE, PE, or DE) per epoch: for an input vector ***x*** = (*x*_1_, *x*_2_, …, *x*_*c*_) representing *c* channel-wise values of a single feature type within one epoch, the transformation produces an output vector of the same length. The macro-scale transformation *g*(*x*) and micro-scale transformation *h*(*x*) operate on each element individually, while the weight parameter *W* is estimated globally from the training set to ensure stability across all samples.

The calculations for the parameters are as follows:

First, the weight parameter *W* is computed from the global statistics of the training set:


$$W=\frac{1}{1+\exp(-a\times \frac{\bar{m}}{\bar{d}})}$$ (5)

where *a* is the sensitivity parameter (set to 2), and $\bar{m}$ and $\bar{d}$ are the mean and standard deviation computed across all feature values of the given feature type in the entire training set. This global estimation ensures consistent scale balancing across the dataset and avoids the instability that would arise from per-epoch estimation with limited channel-wise samples.

Differential entropy under the Gaussian definition changes by log|*c*| when EEG amplitude is rescaled by a factor *c*. Consequently, Equations 4–7, including the mean-to-standard-deviation weight, are not invariant to an arbitrary change from volts to microvolts. We therefore do not claim physical-unit invariance. Instead, the implementation requires EEG amplitudes in volts before DE calculation, and it rejects inconsistent input-unit metadata. All reported analyses used this fixed convention. If the pooled training standard deviation is at most 10^−10^, the mean-to-standard-deviation ratio is set to zero, which gives *W* = 0.5. The logistic argument is clipped to [−20, 20] before exponentiation.

Second, the macroscopic scale transformation function *g*(*x*) applies logarithmic compression to normalize the magnitude of feature values:


g(x)=sign(x)×lg(1+|x|) (6)

Third, the microscopic scale transformation function *h*(*x*) employs robust z-score normalization using the training-set median and interquartile range (IQR):


$$h(x)=\frac{x-\text{median}}{\text{IQR}}$$ (7)

where the median and IQR are estimated from all training-set epochs of the given feature type. If the fitted IQR is at most 10^−10^, it is replaced by 1.0 to avoid division by zero. This fallback maps a constant training feature to zero after centering and prevents numerical amplification of near-constant features. All fitted values are applied unchanged to the held-out fold.

In addition, the MSIRT algorithm augments the fused feature space with cross-feature interaction terms that encode coupling patterns among the three entropy measures:

- FE–DE interactions: Element-wise products between FE and DE in the delta and theta bands (FE × DE_delta_, FE × DE_theta_). These terms encode covariance between time-domain complexity and slow-band variability. Slow-band alterations are commonly reported in AD EEG, although the product terms themselves are methodological features rather than established biomarkers (5, 6).
- PE–DE interaction: Element-wise product between PE and DE in the alpha band (PE × DE_alpha_), encoding how signal randomness covaries with alpha oscillatory activity.

Ratios between differential entropies were removed from revised MSIRT. Differential entropy is logarithmic, so its quotient is neither a spectral-power ratio nor invariant to signal scaling. Removing these terms also eliminates any denominator-near-zero condition.

Before transformation, FE and PE each comprised 8 features and DE comprised 5 × 8 = 40 features, giving 56 dimensions. The transformation preserved these 56 dimensions. The three channel-wise product blocks contributed 24 dimensions, giving 80 dimensions for revised MSIRT. For FE, PE, and the five-band DE block, one median, IQR, mean, standard deviation, and weight *W* were estimated by pooling the relevant values across training epochs and channels. Each DE band used an additional training-fold median and IQR for the product terms. The fitted values were applied unchanged to the held-out fold.

Figure 4 retains the original channel-wise presentation and illustrates the numerical effect of the revised transformation on the FE block for the AD and HC groups. The displayed summaries are descriptive only and are not used as evidence of statistical separation or predictive superiority. The contribution of each transformation component is evaluated separately in the participant-level ablation analysis.

**Figure 4:**
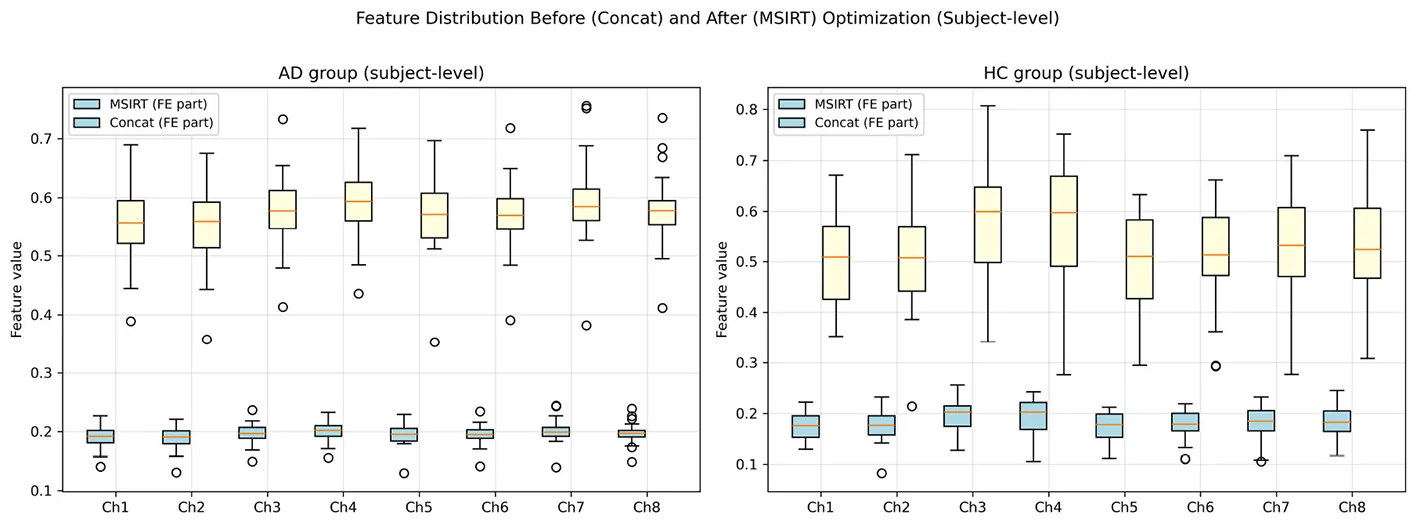
Subject-level FE distributions before direct concatenation and after MSIRT transformation in the AD and HC groups. Each channel is displayed separately. The figure illustrates the numerical effect of the transformation and is not evidence of improved class separability.

Figure 4 illustrates scale compression but is not evidence of improved class separability. Whether the transformation adds predictive information is assessed through the participant-level component ablation.

#### 2.2.4 MSIRT-based classification framework

The evaluation framework comprises derivative-file handling, entropy-feature extraction, MSIRT fusion, classifier training, and subject-level aggregation. RF, support vector machine (SVM), *k*-nearest neighbors (KNN), AdaBoost, and Decision Tree are evaluated as alternative downstream classifiers. Figure 5 shows the RF configuration used for the ablation and permutation analyses, while Figure 6 summarizes the multi-classifier workflow.

**Figure 5:**
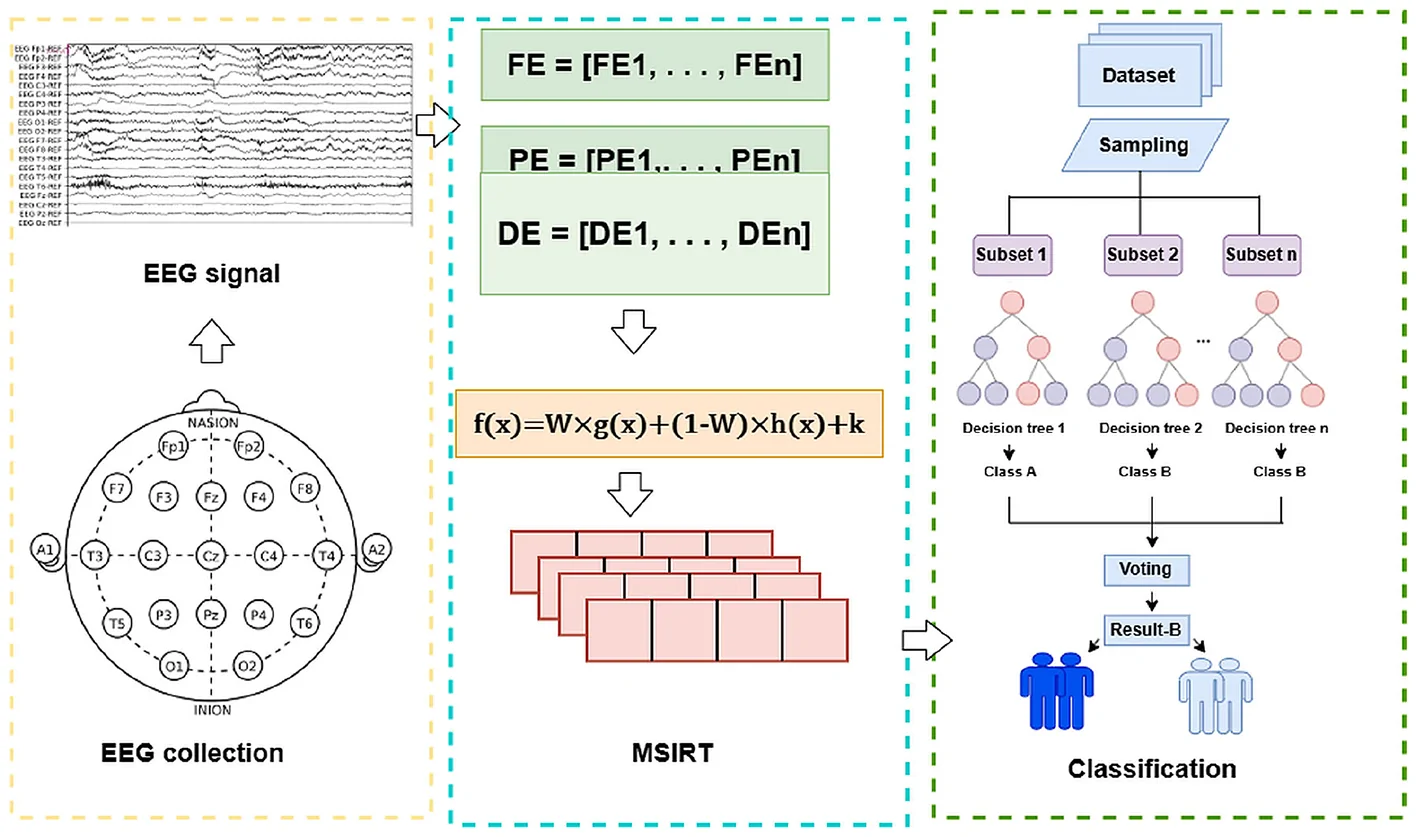
RF configuration of the MSIRT-based classification framework. This configuration was used for the feature-fusion ablation and paired permutation analyses.

**Figure 6:**
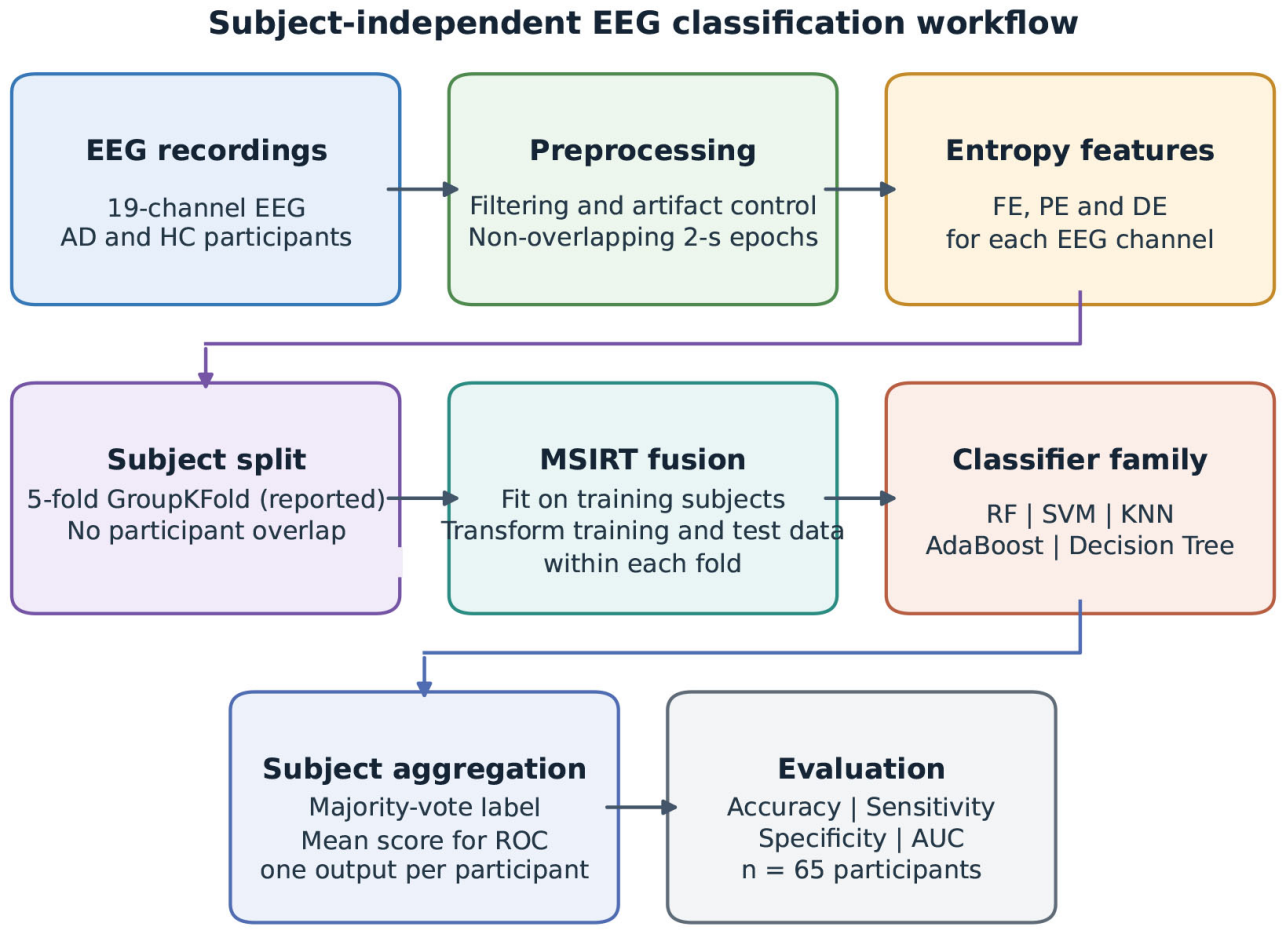
General workflow of the subject-independent pipeline. The reported results use participant-level five-fold cross-validation. Feature transformation and model fitting are restricted to the training participants in each fold.

The Random Forest classifier used 200 estimators, no maximum-depth constraint, the Gini criterion, and random seed 42. These values were fixed for the non-nested comparison and were not tuned on held-out participants. The nested settings are described in the Validation Strategy. The complete revision code, cached 2-s features, split manifest, environment specification, and machine-readable results are supplied as an anonymized code supplement.

#### 2.2.5 Classifier configurations

To determine whether performance depended on the classifier family, MSIRT features were also evaluated using a radial basis function (RBF)-kernel SVM, KNN, AdaBoost, and a Decision Tree. The SVM used *C* = 1.0, gamma=scale, and probability estimation. KNN used *k* = 5, uniform weights, and Euclidean distance. AdaBoost used 100 estimators and a learning rate of 1.0. The Decision Tree used the Gini criterion without a maximum-depth constraint. SVM and KNN were preceded by standardization fitted exclusively on the training portion of each fold. All stochastic classifiers used a random seed of 42, and no classifier was tuned on the held-out test subjects.

### 2.3 Validation strategy

The fixed-model comparisons used participant-level 5-fold GroupKFold cross-validation, with every epoch from one participant confined to either training or testing. The exact fold membership is provided in split_manifest.json. Transformation statistics were fitted only on training-fold epochs. Within each test fold, epoch labels were aggregated by majority vote and probabilities by their arithmetic mean, producing one prediction per participant. Five shuffled, stratified participant-level five-fold partitions were also examined as an internal sensitivity analysis.

Sex-related sensitivity was assessed in two ways. First, the primary out-of-fold predictions were summarized separately for female and male participants. Second, we performed 20 repeated sex-balanced subsamples. Each repetition included all 12 male participants with AD, 11 randomly selected female participants with AD, 12 randomly selected male controls, and all 11 female controls, giving 12 males and 11 females in each diagnostic group (*n* = 46). Within every repetition, the same deterministic 50-epoch selection, participant-level five-fold GroupKFold procedure, RF configuration, training-fold transformation fitting, and subject-level aggregation were applied. The repeated analysis was intended as a sensitivity check and not as a definitive covariate-adjusted estimate.

A separate nested participant-level analysis used five stratified outer folds and three stratified inner folds. Within each outer-training set, the inner analysis selected among concatenation, transformation only, and revised MSIRT. It also selected between Random Forest models with 100 trees, max_features=sqrt, and either unrestricted depth or max_depth=10. The selected representation and model were then fitted to the complete outer-training set and evaluated once on its held-out outer fold. No outer-test participant contributed to representation or model selection. These internal analyses do not replace validation in a well-characterized independent cohort.

## 3 Experiments and results

This section reports an exploratory comparison of five fixed classifier families, followed by a component-wise RF ablation and a paired participant-level comparison between revised MSIRT and direct concatenation.

The experimental procedures of this study are shown in Figure 6. First, the dataset-provided pre-processed EEG derivatives underwent the study-specific channel selection and residual signal checks described above. Subsequently, three types of nonlinear dynamical features were extracted: FE, PE, and DE. On this basis, the MSIRT algorithm was introduced to fuse and optimize these features, thereby constructing a feature representation. The dataset was then evaluated using subject-level 5-fold GroupKFold cross-validation as described in the Validation Strategy section. The overall framework comprises derivative-file handling, feature extraction and fusion, model training, and performance evaluation.

### 3.1 Residual quality control

Across all 65 participants, 25,664 of 26,591 2-s epochs were retained after residual quality control. The AD group retained 14,091 of 14,550 epochs, with a mean rejection fraction of 3.00% and a median of 2.14%. The HC group retained 11,573 of 12,041 epochs, with a mean rejection fraction of 3.80% and a median of 2.71%. Five epochs exceeded the 500μV threshold, and no epoch contained nonfinite values or met the flat-segment rule. Most exclusions were triggered by the recording-relative high-noise rule.

The mean retained counts were 391.42 ± 78.43 epochs for AD and 399.07 ± 30.92 for HC. Group differences were not detected for either rejection fraction (two-sided Mann–Whitney *p* = 0.6582) or retained epoch count (*p* = 0.6300). No participant was excluded from the subsequent analyses.

### 3.2 Comparison of machine learning classifiers

To assess classifier dependence, RF, SVM, KNN, AdaBoost, and Decision Tree were compared using the same 2-s MSIRT features, the same five subject-level GroupKFold splits, and the same subject-level aggregation procedure. RF was subsequently held fixed for the component ablation and paired permutation analysis to isolate changes in feature representation while keeping the classifier constant.

Figure 7 and Table 2 present the exploratory classifier comparison. Accuracy ranged from 78.46% to 86.15%, and the area under the ROC curve (AUC) ranged from 0.8836 to 0.9090. SVM produced the highest numerical AUC, while RF produced the highest accuracy. The overlapping bootstrap intervals do not support a definitive classifier ranking.

**Figure 7:**
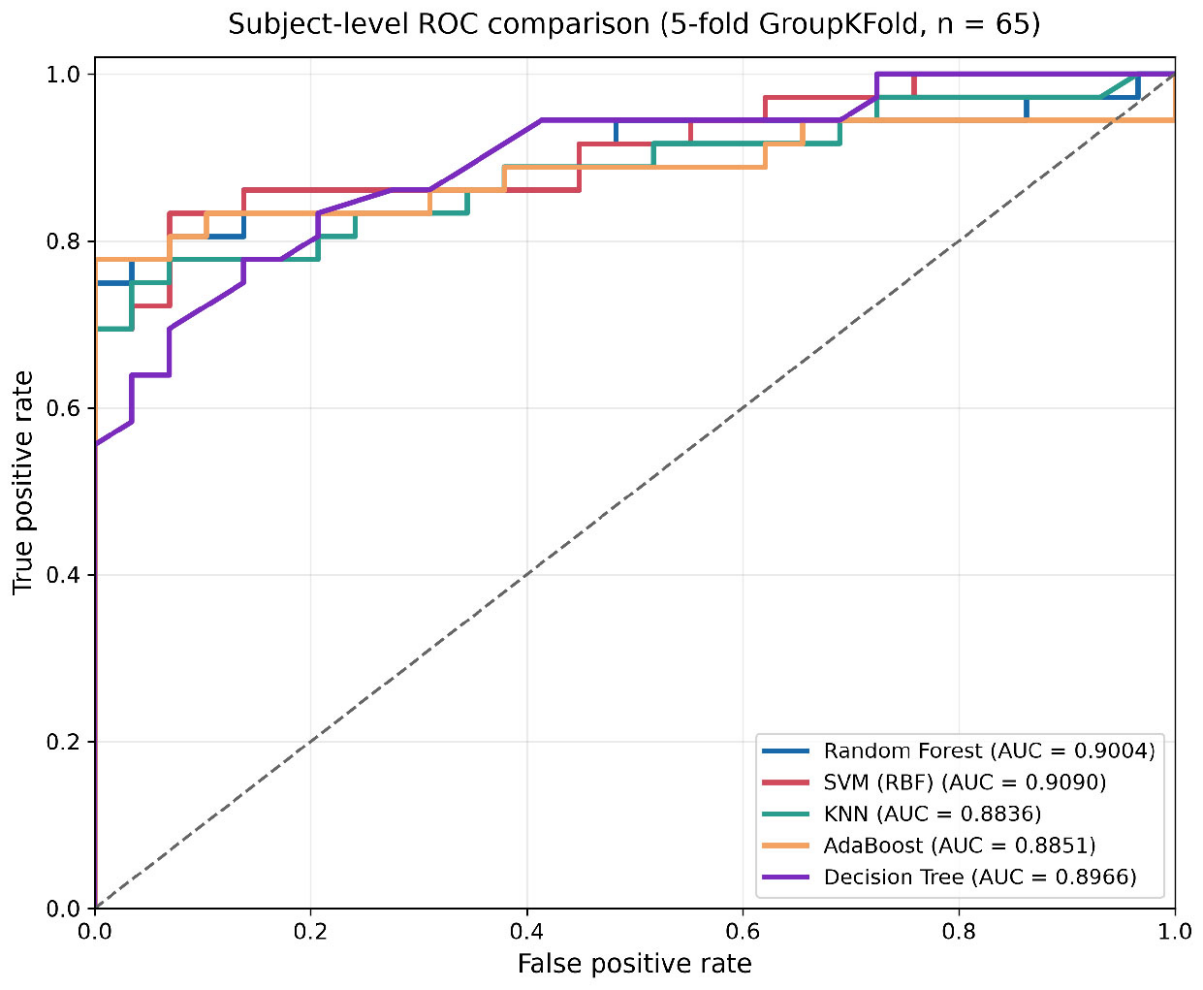
Subject-level receiver operating characteristic (ROC) curves of five classifiers using MSIRT features under five-fold GroupKFold cross-validation. Each curve was calculated from one aggregated probability per participant (*n* = 65).

**Table 2:** Classifier performance with MSIRT features under subject-level 5-fold cross-validation.

Classifier	Acc (%)	Spe (%)	Sen (%)	AUC	Fold AUC	*p* vs. RF
RF	86.15	89.66	83.33	0.9004	0.9126 ± 0.0856	–
SVM (RBF)	84.62	86.21	83.33	0.9090	0.9324 ± 0.0550	0.375
KNN	80.00	86.21	75.00	0.8836	0.8911 ± 0.1093	0.438
AdaBoost	78.46	72.41	83.33	0.8851	0.9031 ± 0.0983	1.000
Decision tree	80.00	79.31	80.56	0.8966	0.9126 ± 0.0835	0.750

Fold AUC is reported as mean ± standard deviation. The *p*-values are from two-sided Wilcoxon signed-rank tests of the five fold-level AUCs against RF.

None of the paired fold-wise AUC comparisons differed significantly from RF (all *p*≥0.375). These 5-fold tests have limited power and are reported descriptively.

The subject-level bootstrap 95% AUC intervals were [0.8103, 0.9713] for RF, [0.8266, 0.9722] for SVM, [0.7864, 0.9569] for KNN [0.7873, 0.9674] for AdaBoost, and [0.8156, 0.9622] for Decision Tree.

### 3.3 Nested participant-level validation

The nested analysis selected revised MSIRT in all five outer folds. Four folds selected the unrestricted-depth Random Forest, and one selected max_depth=10. The pooled outer-fold predictions yielded 87.69% accuracy and an AUC of 0.9119. Outer-fold accuracies were 92.31%, 92.31%, 100.00%, 76.92%, and 76.92%; the corresponding AUCs were 1.0000, 0.9286, 1.0000, 0.9286, and 0.7381. The spread across outer folds shows the uncertainty expected from a cohort of 65 participants.

### 3.4 Epoch definition

The 2-s epoch length was fixed for the revised analysis rather than selected using held-out performance. Earlier comparisons of 2-, 3-, and 4-s epochs were removed from the main analysis because they used the same dataset for methodological comparison and reporting.

The fixed 50-epoch selection was compared with 20 random selections and with aggregation over all valid epochs. For random 50-epoch selections, concatenation yielded an accuracy of 82.77%±3.14% and an AUC of 0.8850 ± 0.0111. Revised MSIRT yielded an accuracy of 83.31%±2.70% and an AUC of 0.8882 ± 0.0107. The respective AUC ranges were 0.8592–0.9023 and 0.8649–0.9080. Using all valid epochs, concatenation achieved 80.00% accuracy and an AUC of 0.8649, while revised MSIRT achieved 83.08% accuracy and an AUC of 0.8736.

The deterministic selection produced AUCs near the upper end of the random-selection distributions. This sensitivity analysis therefore indicates that AUC varies with the selected epoch subset. The fixed analysis is retained as the pre-specified primary representation, while the repeated and all-epoch estimates quantify this sampling dependence.

### 3.5 Component ablation

Five representations were compared with RF under identical participant splits: direct concatenation, robust normalization only, the full weighted transform without products, robustly normalized base features with the three product blocks, and revised MSIRT. The fourth representation isolates the interaction products without the weighted signed-log transform. The entropy cache, participant splits, classifier, and aggregation rule were unchanged. Robust normalization and the weighted no-product transform are feature-wise strictly monotonic under the fitted numerical safeguards. They therefore preserve within-feature ordering and were treated as RF invariance controls rather than as transformations that introduce new predictive information. The corresponding results are summarized in Table 3.

**Table 3:** Component-wise ablation with RF under participant-level 5-fold cross-validation.

Representation	Dimensions	Acc (%)	Spe (%)	Sen (%)	AUC
Concatenation	56	84.62	86.21	83.33	0.8956
Robust normalization only	56	84.62	86.21	83.33	0.8956
Full transform, no products	56	86.15	86.21	86.11	0.8956
Robust base + products	80	80.00	79.31	80.56	0.8908
Revised MSIRT	80	86.15	89.66	83.33	0.9004

Robust normalization reproduced the concatenation metrics, as expected for a feature-wise monotonic remapping. The weighted no-product transform also preserved AUC; its one-participant accuracy difference is not interpreted as new information because RF split order is unchanged by a strictly monotonic feature transformation. Adding the interaction products to the robust base reduced accuracy to 80.00% and AUC to 0.8908. The complete MSIRT representation achieved 86.15% accuracy and an AUC of 0.9004. Thus, the analysis did not demonstrate a stable independent benefit from the interaction products or from the feature-wise transform. It only showed that their combined numerical representation behaved differently in this finite RF implementation.

### 3.6 Sex-related sensitivity analysis

The sex distribution differed between diagnostic groups (AD: 24 female and 12 male; HC: 11 female and 18 male; Fisher's exact odds ratio 3.27, two-sided *p* = 0.0262). In the primary split, revised MSIRT achieved an AUC of 0.8788 in female participants (*n* = 35) and 0.9769 in male participants (*n* = 30); the corresponding accuracies were 80.00% and 93.33%. These sex-stratified estimates are imprecise because each stratum contains few participants and unequal diagnostic counts.

Across 20 sex-balanced resamples (*n* = 46 per repetition), direct concatenation achieved a mean accuracy of 80.00% (SD 4.66%) and a mean AUC of 0.8709 (SD 0.0456). Revised MSIRT achieved a mean accuracy of 81.74% (SD 4.26%) and a mean AUC of 0.8846 (SD 0.0459). The empirical 95% intervals for revised MSIRT were 76.09–89.24% for accuracy and 0.8156–0.9609 for AUC. The mean AUC difference from concatenation was 0.0138. Performance therefore remained above chance after balancing sex, but the lower and more variable estimates indicate that sex composition may have contributed to the full-cohort results. This sensitivity analysis reduces but does not eliminate concern about demographic confounding.

### 3.7 Paired AUC comparison

Revised MSIRT and concatenation produced paired out-of-fold probabilities for the same 65 participants. The AUC difference was assessed with 10,000 stratified paired bootstrap resamples and 10,000 paired permutations in which the two method probabilities were exchangeable within participant. The AUC difference (revised MSIRT minus concatenation) was 0.0048, with a bootstrap 95% confidence interval of −0.0067 to 0.0192 and a two-sided paired permutation *p*-value of 0.3780. Revised MSIRT therefore produced a small numerical AUC increase, but the participant-level comparison did not establish statistical superiority.

## 4 Discussion

Prior studies have evaluated nonlinear resting-state EEG features for participant classification in AD (7, 11). The present study evaluates MSIRT as a classifier-independent feature-fusion framework under participant-level validation. The experiments assess both the feature representation and its behavior across five classical classifier families.

The present analysis is limited to classical FE, PE, and DE descriptors. Recent methods include amplitude-adjusted multiscale FE (12) and Multivariate Multiscale Multi-Frequency Entropy (M3FrEn), which jointly represents temporal, spatial, and spectral EEG properties (18). These methods were not evaluated here, and no comparative claim is made.

In this study, FE, PE, and DE were selected to represent time-domain complexity, sequential ordinal structure, and band-limited log variance, respectively. No claim about the performance of an individual feature family is made because the revised study did not report a pre-specified single-feature comparison.

The component ablation separates effects that were previously combined under the MSIRT label. Feature-wise robust normalization and the weighted no-product transform preserve the order of each feature and therefore cannot be interpreted as introducing new split information for RF. The interaction-only representation did not improve performance, whereas the complete representation produced a small numerical AUC difference from concatenation. These findings do not isolate a stable benefit from either the monotonic transform or the interaction products. Ratios between differential entropies were removed because they lacked a valid spectral interpretation. Across five repeated participant partitions, revised MSIRT showed a higher mean AUC than concatenation (0.9033 vs. 0.8914), but the paired confidence interval for the primary split included zero.

The current weighting rule is conditional on the fixed voltage convention. Because Gaussian DE shifts additively under amplitude rescaling, the signed logarithmic term and *W* are not invariant to arbitrary physical units. Standardizing all inputs to volts makes the reported implementation reproducible, but it does not provide mathematical unit invariance. A future formulation should replace the location-dependent weight with a dimensionless statistic before evaluation in an independent cohort.

A strength of this study is the use of the public OpenNeuro ds004504 dataset. No statistically significant mean age difference was detected between the AD and HC groups (AD: 66.4±7.9 years; HC: 67.9±5.4 years; *p* = 0.38). By contrast, sex was imbalanced between groups. Sex-stratified performance differed, and sex-balanced resampling produced lower mean AUCs than the primary full-cohort analysis. These observations are compatible with partial demographic confounding, although they do not quantify a causal sex effect. The small cohort and the association between sex and diagnosis prevent definitive covariate adjustment.

The five fixed classifier families produced AUCs from 0.8836 to 0.9090. Their bootstrap intervals overlapped substantially, and the five folds are not independent experimental replications. The comparison is therefore descriptive rather than evidence for a classifier ranking. A sensitivity analysis using five shuffled, stratified participant-level five-fold partitions gave mean AUCs of 0.8914 (direct concatenation), 0.8912 (transformation only), and 0.9033 (revised MSIRT), with standard deviations of 0.0125, 0.0126, and 0.0124, respectively.

The revised study fixed the epoch length at 2 s and used deterministic, equally spaced epoch selection. Across 20 random selections, accuracy was comparatively stable, but AUC varied with the chosen subset. Aggregation over all valid epochs also reduced AUC for both representations. The deterministic selection was near the upper end of the random AUC distribution and should not be interpreted as an optimal sampling rule.

The single public cohort, limited participant count, eight-channel analysis, and reliance on dataset-provided pre-processing remain important limitations. We performed no additional filtering, rereferencing, artifact-component removal, or channel interpolation. The residual quality-control analysis did not detect unequal retention between AD and HC, but it cannot establish that every remaining epoch is free of ocular, muscle, or movement activity.

Accordingly, this work should be interpreted as an exploratory evaluation of entropy-feature representations rather than a clinical diagnostic validation. Confirmatory work requires larger, well-characterized independent cohorts and validation procedures that incorporate model and epoch-sampling uncertainty.

## 5 Conclusion

This exploratory study revised MSIRT by removing ratios between differential entropies and fitting every normalization parameter within the training fold. With RF fixed, revised MSIRT achieved 86.15% accuracy and an AUC of 0.9004, compared with 84.62% and 0.8956 for direct concatenation. The paired AUC difference was 0.0048 (95% confidence interval, −0.0067 to 0.0192; *p* = 0.3780). Nested participant-level selection achieved 87.69% accuracy and an AUC of 0.9119, with revised MSIRT selected in all five outer folds.

The findings show a small numerical difference for the complete representation, but neither the component ablation nor the sex-balanced sensitivity analysis establishes a stable independent contribution, statistical superiority, or clinical utility. Confirmatory assessment requires larger, demographically balanced independent cohorts and validation procedures that incorporate method and epoch-selection uncertainty.

## Data Availability

The EEG dataset analyzed in this article is publicly available in OpenNeuro as ds004504, version 1.0.0 (dataset DOI: 10.18112/openneuro.ds004504.v1.0.0), under the dataset's stated public-use terms. The revised submission includes an anonymized code supplement containing the analysis-ready entropy-feature cache, corrected MSIRT implementation, exact participant split manifest, sex-sensitivity and component-ablation scripts, environment requirements, and machine-readable results. The supplied code begins from the cached features used in the reported experiments; the dataset-provided preprocessing is documented rather than reimplemented.
